# The Retrievability of Calcium Silicate-Based Sealer during Retreatment and the Effectiveness of Additional Passive Ultrasonic Irrigation: A Microcomputed Tomographic Study

**DOI:** 10.1155/2022/3933305

**Published:** 2022-01-24

**Authors:** TaeYeon Lee, Se Hoon Kahm, KyungJae Kim, SungEun Yang

**Affiliations:** ^1^Department of Conservative Dentistry, Yeouido St. Mary's Hospital, College of Medicine, The Catholic University of Korea, Seoul 07345, Republic of Korea; ^2^Department of Dentistry, Eunpyeong St. Mary's Dental Hospital, College of Medicine, The Catholic University of Korea, Seoul 03312, Republic of Korea; ^3^Private Dental Clinic, Republic of Korea; ^4^Department of Conservative Dentistry, Seoul St. Mary's Dental Hospital, College of Medicine, The Catholic University of Korea, Seoul 06591, Republic of Korea

## Abstract

This study investigated the retreatability of EndoSeal MTA (Maruch, Wonju, Korea) according to the presence or absence of a canal isthmus and the additional use of passive ultrasonic irrigation (PUI) through microcomputed tomography (micro-CT) imaging. An epoxy resin sealer (AH Plus (Dentsply DeTrey, Konstanz, Germany)) was used as a reference for comparison. Forty-five artificial mandibular molars (TRUETOOTH #19, DELABS, Santa Barbara, CA) with a mesial canal with an isthmus and a distal canal without an isthmus were obturated using gutta-percha and one of the following sealers (*n* = 15 each): AH Plus, EndoSeal MTA, and EndoSeal MTA + PUI. Micro-CT scanning was performed to assess the void volume (as a percentage) at three root levels. After the root fillings were removed, second micro-CT scanning was conducted to evaluate the amount of remaining root filling material. The Kruskal-Wallis *H* test and post hoc analysis were used for between-group comparisons. The Mann–Whitney *U* test was used for comparisons between canals with and without an isthmus (*p* < 0.05). In the EndoSeal MTA group, the void volume and remaining filling materials were higher irrespective of the presence or absence of an isthmus. In apical lesions in the EndoSeal MTA group, the void ratio was significantly lower, and there was a significantly higher amount of remaining filling material. Regardless of the presence of an isthmus, the amount of remaining filling material of the EndoSeal + PUI group was reduced to a similar degree as the AH plus group. When performing retreatment for root canals filled with EndoSeal MTA, removal of the filling material can be more difficult in the apical region. The additional use of PUI can improve the efficacy of removal.

## 1. Introduction

Periapical disease might not be resolved even following root canal treatment if there is a persistent bacterial infection or if infection recurs [1, 2]. In nonsurgical endodontic retreatment, the root canal filling is completely removed to access the apical foramen, through which the root canal system is cleaned and shaped [3, 4]. This process necessitates removing filling materials that may serve as a bacterial reservoir and providing an environment that promotes healing [5]. Incomplete removal of filling material from the walls of the canal can hinder these goals, as the remaining material may contain microbes.

Materials based on calcium silicate, such as EndoSeal MTA (Maruch, Wonju, Korea), EndoSequence BC Sealer (Brasseler USA, Savannah, GA, USA), and MTA Fillapex (Angelus, Londrina, Brazil), have recently been developed. These materials are minimally cytotoxic, have an appropriate bonding strength and sealing ability, and have been reported to stimulate biomineralization [6]. EndoSeal MTA is composed of calcium silicate, calcium sulfate, and calcium aluminate, as well as a radiopacifier, and can be set in humid conditions, such as those encountered in root canal systems. The available formulations of EndoSeal MTA include a premixed, ready-to-use product, and an injectable paste [7]. Previous research has confirmed that EndoSeal MTA has appropriate physical properties, high biocompatibility, and a high degree of bond strength, as well as showing minimal discoloration and a favorable distribution of sealer [8].

Since materials containing MTA harden upon setting and have the ability to undergo biomineralization, concerns have been raised regarding the retrievability of canals obturated with MTA-based sealers [5]. A primary requirement for a root canal sealer, in addition to its sealing properties, is removability. Although numerous studies have investigated the sealing ability of calcium silicate-based sealers, fewer studies have explored their retrievability, with considerable inconsistency in their findings [9, 10].

Passive ultrasonic irrigation (PUI) is used for the removal of bacteria, dental debris, and the smear layer [11, 12]. Its mechanism involves acoustic streaming and cavitation in the irrigation solution applied to the root canal. In the context of retreatment, previous studies have found that PUI improved sealer removal [13–15]. Nonetheless, no studies have yet investigated the effectiveness of PUI in combination with nickel-titanium (NiTi) reinstrumentation for the removal of gutta-percha (GP) and residual tricalcium silicate-based sealer.

This study used microcomputed tomography (micro-CT) to compare the residual filling materials of EndoSeal MTA and AH Plus after mechanical reinstrumentation and to evaluate whether the additional use of PUI improved the efficacy of EndoSeal MTA removal.

## 2. Materials and Methods

### 2.1. Sample Preparation

**Figure 1:**
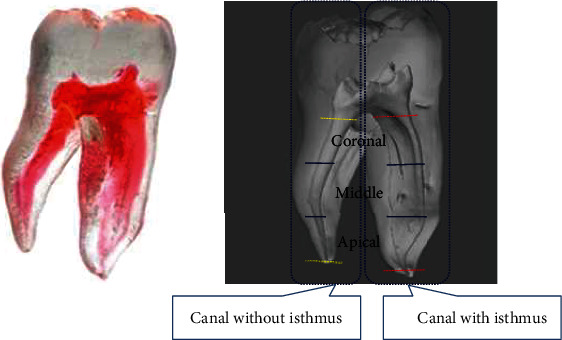
An artificial mandibular molar with an isthmus in the mesial canal.

The study received approval from the Institutional Review Board of Seoul St. Mary's Hospital, Catholic University of Korea (KC16EISI0332). Forty-five artificial mandibular molar teeth (TRUETOOTH #19, DELABS, Santa Barbara, CA, USA, Figure 1) with an isthmus between the mesio-lingual and mesio-buccal canals were utilized. In order to increase the reliability of results, the process of root canal filling and retreatment was performed by one endodontist, and then, cbct observation and analysis were performed by two endodontists, and the average value was obtained as data.

### 2.2. Root Canal Preparation

A #4 round bur and an Endo-Z bur (Dentsply Maillefer, Ballaigues, Switzerland) were used to prepare an access cavity in each tooth. A K-file (size 10; Dentsply Maillefer) was inserted into the root canal until it was observable at the apex, and the working length (WL) was calculated as this distance minus 1 mm. Instrumentation of the root canals was performed using an X-Smart electronic motor (Dentsply Maillefer) and ProFile system (Dentsply Maillefer) via a crown-down sequence, with a master apical file (MAF) size of #35/0.04 in the distal canals and #30/0.04 in mesial canals. Then, 1 mL of 3% NaOCl was administered for canal irrigation in intervals between file application. The final irrigation utilized 1 mL of NaOCl and 1 mL of 17% EDTA for 1 min, with the subsequent application of 5 mL of NaOCl. A plastic syringe with a 27-G needle (Monoject endodontic needle, Tyco/Kendall, Mansfield, MA, USA) was inserted without reaching the binding point. After using paper points (Dentsply Maillefer) to dry the root canal walls, master GP cones (Diadent, Cheongju, Korea) were chosen in accordance with each canal's MAF size, and their fit was evaluated by assessing the tug-back sensation.

### 2.3. Root Canal Obturation

Teeth were randomly assigned to three groups. In the AH Plus group, teeth were obturated using the continuous wave technique with GP and a resin-based sealer (AH Plus, Dentsply Maillefer), while in the EndoSeal MTA groups and EndoSeal + PUI group, teeth were obturated with the single-cone technique using GP and a calcium silicate–based sealer (EndoSeal MTA, Maruchi, Wonju, Korea). The master cones in the AH Plus group were coated with AH Plus sealer and placed at a suitable length. Root canal obturation was performed using the continuous wave technique (System B; SybronEndo, Orange, CA, USA and Obtura; Obtura Spartan, Fenton, MO, USA). Root canal obturation in the EndoSeal MTA groups was performed in accordance with the manufacturers' instructions. Injection tips were used to deliver the sealers. The master cones were inserted and pumped twice or thrice. The master cones were then cut at the level of the orifice using System B (SybronEndo Orange, CA, USA). Temporary sealing of the access cavities was performed using Caviton (GC, Tokyo, Japan), and all samples were placed in a storage chamber under conditions of 100% relative humidity and a temperature of 37°C for 15 days to ensure that the sealer hardened [16, 17].

### 2.4. Micro-CT Imaging to Measure the Filling Material

A micro-CT scanner (Skyscan 1172, Bruker micro-CT, Kontich, Belgium) was used to scan the teeth before and after retreatment, with settings of a voxel dimension of 23.86 *μ*m, a source voltage of 100 kV, and a current of 100 *μ*A. A 0.5 mm thick aluminum filter was used with rotational steps of 0.83° increments for a total rotation of 180°. Images obtained from the scan were reconstructed using NRecon (Version 1.14.4.1, Bruker micro-CT). Two-dimensional horizontal slices were obtained at 3 mm above the apex (apical), 2 mm below the cemento-enamel junction (coronal), and at the midpoint between the apical and coronal points (middle) using Dataviewer (ver. 1.5.0, Bruker micro-CT). Pixelation of the images was performed using CT-An (ver.1.14.4.1, Bruker micro-CT). The mesial root and distal root were imaged differently, based on the root axis. To evaluate the overall filling state, three-dimensional (3D) images of the filling material were visualized by the surface-rendering program CT-Vol (SkyScan). The root canal space was removed, and the volume of filling materials and voids was calculated before retreatment in the apical, middle, and coronal slices [18]. The CT-An software (SkyScan) was used to measure the volume of the gap between the filling material and root canal walls and the voids in the filling material. Three-dimensional image data were obtained after obturation in the *x*, *y*, and *z* axes for the mesial and distal root axes. When measuring the voids between the filling material and the root canal wall, a gray scale ranging between 40 and 255 was assigned as the volume of the filling material, and a gray scale ranging between 0 and 40 was assigned as a void. When measuring voids inside the filling material, a gray scale ranging between 124 and 255 was assigned as the volume of the filling material, and a gray scale ranging between 0 and 124 was assigned as a void. We calculated the percentage area of voids of filling materials using the equation below.

(Volume of voids of filling material)/(Volume of filling material) × 100 = Percentage (%) of voids of filling material.

### 2.5. Removal of the Root Filling Materials and Reinstrumentation

After 15 days, the root canals were instrumented using Gates Glidden drills (#2, #3, and #4) for removal of the root canal filling materials. The ProFile system was utilized, with MAF sizes of #35/0.06 in mesial canals and #40/0.06 in distal canals. In the retreatment, the MAF was 1 size higher than that used in the first treatment. Then, 1 mL of 3% NaOCl was used for canal irrigation between file applications, and the final irrigation was conducted using 1 mL of NaOCl and 1 mL of 17% EDTA for 1 min. The final step was the application of 5 mL of NaOCl. A plastic syringe with a 27-G needle (Monoject endodontic needle, Tyco/Kendall, Mansfield, MA, USA) was inserted, without reaching the binding point. PUI was additionally used in group 3.

### 2.6. Micro-CT Imaging to Measure the Remaining Filling Material

**Figure 2:**
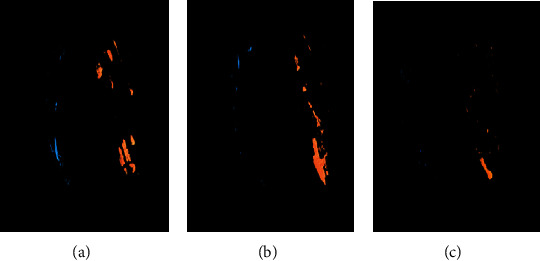
Micro-CT reconstructed images of the remaining filling materials. Blue color indicates the remaining filling materials in the canal without isthmus, and orange indicates the remaining filling materials in the canal with isthmus: (a) AH Plus group; (b) EndoSeal MTA group; (c) EndoSeal MTA + PUI group.

The second micro-CT scans used the same settings as the initial scan.  Figure 2 presents micro-CT images of the residual filling materials, which were measured at the apical, middle, and coronal thirds. The percentage volume of residual materials was calculated using the following equation for each sealer type and position of the horizontal section.

(Volume of residual filling material following retreatment)/(Volume of initial filling material) × 100 = Percentage (%) of remaining filling material.

### 2.7. Statistical Analysis

All statistical analyses were conducted using R version 3.3.3 (R Foundation for Statistical Computing, Vienna, Austria) and T&F version 3.0 (YooJin BioSoft, Korea). The variables were presented as median (interquartile range). To compare the filling ratio (%) or remaining ratio (%) among the AH Plus, EndoSeal MTA, and EndoSeal MTA + PUI groups, the Kruskal-Wallis *H* test was performed. In all treatment groups, comparisons among the apical, middle, and coronal regions were made with the Kruskal-Wallis *H* test. Comparative analyses between canals with and without an isthmus were performed using the Mann–Whitney *U* test. The Bonferroni correction was used for a post hoc analysis after the Kruskal-Wallis *H* test. In all analyses, a *p* value of < 0.05 was considered indicative of statistical significance.

## 3. Results

**Figure 3:**
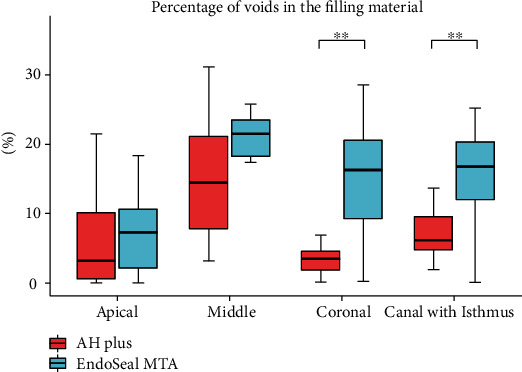
Distribution of the percentage of voids in the filling material in canals with an isthmus between the AH Plus group and the EndoSeal MTA group. Data are presented as median (interquartile range). *p* values were computed using the Mann–Whitney *U* test. ^∗^*p* < 0.05, ^∗∗^*p* < 0.01, ^∗∗∗^*p* < 0.001.

**Figure 4:**
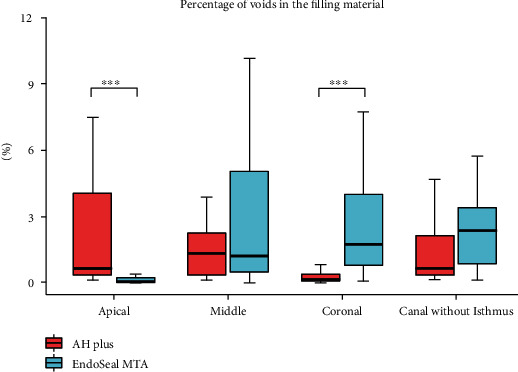
Distribution of percentage of voids in the filling material in canals without an isthmus between the AH Plus group and the EndoSeal MTA group. Data are presented as median (interquartile range). *p* values were computed using the Mann–Whitney *U* test. ^∗^*p* < 0.05, ^∗∗^*p* < 0.01, ^∗∗∗^*p* < 0.001.

Figure 3 (canal with isthmus) and Figure 4 (canal without isthmus) show measurements of the void ratios according to the location of root canal filling. The void ratios were higher in the root canals with an isthmus in both groups (AH Plus: 6.16% vs. 0.65%, EndoSeal MTA: 16.79% vs. 2.37%). Irrespective of the presence or absence of an isthmus, the void ratio was higher in the EndoSeal MTA group (with isthmus: 6.16% vs. 16.79%, without isthmus: 0.65% vs. 2.37%). In the analysis according to the location of the root canal, the void ratio of the EndoSeal MTA was significantly higher than that of the AH Plus group in the coronal region irrespective of the presence or absence of an isthmus (with isthmus: 3.48% vs. 16.27%, without isthmus: 0.14% vs. 1.73%). In the apical region of root canals without an isthmus, the void ratio was significantly lower in the EndoSeal MTA group (0.02%) than in the AH Plus group (0.63%). In the AH Plus group, the void ratios were low in the coronal region irrespective of the presence or absence of an isthmus (3.48% vs. 0.14%). In the EndoSeal MTA group, the void ratios were low in the apical region irrespective of the presence or absence of an isthmus (7.26% vs. 0.02%).

**Figure 5:**
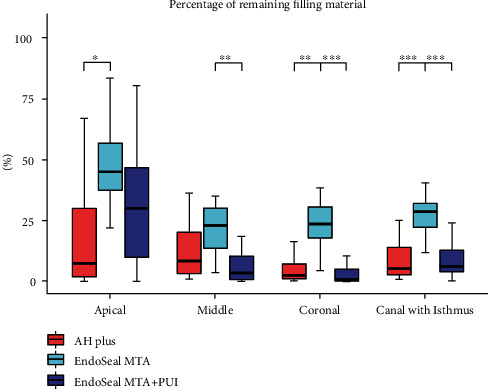
Distribution of the remaining ratio in canals with an isthmus among the AH Plus, EndoSeal MTA, and EndoSeal MTA + PUI groups. Data are presented as median (interquartile range). *p* values were adjusted using the Bonferroni post hoc method. ^∗^*p* < 0.05, ^∗∗^*p* < 0.01, ^∗∗∗^*p* < 0.001.

The remaining ratios according to the position of the root canals with an isthmus are shown in Figure 5 and Table 1. The highest remaining ratio was observed in the EndoSeal MTA group (28.47%), and no significant difference was observed between the AH Plus group (5.12%) and the EndoSeal+PUI group (6.06%). However, the remaining ratio was significantly lower in the EndoSeal MTA + PUI group (6.06%) than in the EndoSeal MTA group (28.47%). In the coronal and middle regions, significantly lower remaining ratios were observed in the EndoSeal MTA + PUI group than in the EndoSeal MTA group, but no significant difference was observed in the apical region. In the AH Plus group, there was no difference according to the location of the root canal (7.46% apical); however, the remaining ratios were significantly higher in the apical region compared to other locations in the EndoSeal MTA group (45.14%) and the EndoSeal MTA + PUI group (30.13%).

**Figure 6:**
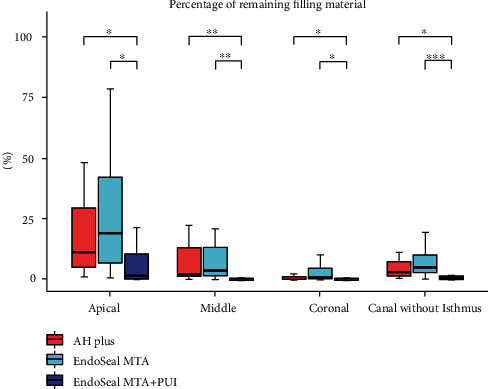
Distribution of the remaining ratio in canals without an isthmus among the AH Plus, EndoSeal MTA, and EndoSeal MTA + PUI groups. Data are presented as median (interquartile range). *p* values were adjusted using the Bonferroni post hoc method. ^∗^*p* < 0.05, ^∗∗^*p* < 0.01, ^∗∗∗^*p* < 0.001.

The remaining ratios according to the position of the root canals without isthmus are shown in Figure 6 and Table 2. The highest remaining ratio was observed in the EndoSeal MTA group (4.96%), and a significantly lower remaining ratio was observed in the EndoSeal MTA + PUI group (0.32%). Significantly lower remaining ratios were observed in all positions when PUI was additionally used. The remaining ratios were significantly higher in the apical region than in the other positions in the AH Plus group (11.57%) and the EndoSeal MTA group (18.97%), while no difference was observed according to location in the EndoSeal MTA + PUI group (1.26% apical).

Table 3 shows the results of a comparison of the remaining ratios according to the presence or absence of isthmus in the root canals for each group. In the AH Plus group, there was no significant difference in the remaining ratio according to the presence or absence of an isthmus (5.12% vs. 2.86%). The remaining ratios were significantly higher in the root canals with isthmus in the EndoSeal MTA group (28.47% vs. 4.96%) and in the EndoSeal MTA + PUI group (6.06% vs. 0.32%), and significant differences were observed in all locations of the canals.

## 4. Discussion

In the current study, artificial teeth were used. Using natural teeth for experiments may have the advantage of reproducing the clinical setting, but the extensive morphological variation in root canals results in difficulty establishing between-group standardization. In contrast, artificial teeth have the advantage of enabling the radius, location, and degree of root canal curvature to be standardized [15].

In the comparison of void ratios between AH Plus and EndoSeal MTA, it was found that AH Plus showed a high void ratio in the apical third, while EndoSeal MTA had a void ratio in the coronal third. The continuous wave technique was used for AH Plus, and the one-cone technique was used for EndoSeal MTA. The high coronal void ratio in the EndoSeal-filled root canals may be attributed to the use of the one cone technique, wherein a larger canal volume corresponds to a higher sealer-filled volume, thereby increasing the possibility of void formation. In the apical region, the void ratio was lower in the EndoSeal MTA group than in the AH Plus group. A previous study reported that EndoSeal MTA has the advantage of being able to fit into a complex structure [19]. We interpret these findings as indicating that in the apical region, where the volume of the root canal is smaller and the shape is more complex; the one cone technique involving the use of a sealer to fill the complicated part of the root canal can be more advantageous than the thermoplastic condensation method, in which the GP cone fills the complicated part of the root canal.

The highest remaining ratios were observed in the EndoSeal MTA group for both the root canals with and without an isthmus; in particular, significantly higher remaining ratios were observed in the apical third. In the AH Plus group, there was no significant difference in the remaining ratio according to the presence or absence of an isthmus, whereas in the EndoSeal MTA group and EndoSeal MTA + PUI group, the remaining ratio was significantly higher in the root canals with an isthmus. Previous studies also reported the insufficient removability of EndoSeal MTA [20]. It was found that EndoSeal MTA cannot be used as a routine ortho-grade root canal filling material because of its sandy and irretrievable properties, and additional care should be taken when used in a complicated root canal system [20]. This behavior may also be linked to the properties of MTA when it comes into contact with dentin; in particular, MTA can form carbonated apatite or hydroxyapatite when it encounters fluid in a dentinal tubule. These mechanisms enable the formation of chemical bonds between dentinal walls and MTA [21]. For complex root canal systems, the possibility of retreatment should be taken into consideration if EndoSeal MTA is used as a root canal sealer.

The amount of residual filling material was lower in the EndoSeal MTA + PUI group, with the exception of the apical region in canals with an isthmus. It has been reported that PUI use improved the efficiency of dentin debris removal in the root canal [22]. Other studies reported that there was no significant difference between AH Plus and EndoSeal when PUI was used additionally for EndoSeal removal [23]. The lower removal efficiency with the use of PUI in the apical third was similar to that of a previous study [24]. It has been proposed that ultrasound tips are maximally effective in the coronal and middle regions due to limitations of tip movement as it passes through the curvature [25]. The concomitant use of PUI and a cleaning solution strengthens the flushing action of irrigants and speeds up their flow [26, 27]. It is thought that this capacity of PUI contributed to increasing the efficiency of EndoSeal MTA removal from the root canal.

The results of each study should be interpreted with due consideration of the methodology utilized to assess the remaining filling material. Earlier research investigating the retreatability of root filling materials conducted longitudinal tooth sectioning with subsequent digital surface imaging or scanning electron microscopy. A disadvantage of these methods is that they are two-dimensional and do not allow accurate measurements to be made of the quantity of root filling that remains present inside root canals [21]. In this study, we used micro-CT to quantitatively assess the amount of remnants of filling materials. The advantages of micro-CT lie in its nondestructiveness, precision, and reproducibility; furthermore, it provides reconstructions in 3 dimensions and makes it possible to quantitatively evaluate the residual filling material, sealer, and dentin as separate substances on the canal wall pre- and post-retreatment [28].

All specimens in this study showed residual sealer after the removal trial, in alignment with prior research [29, 30]. Apical patency was achieved in all specimens, but the EndoSeal MTA group had a significantly higher remaining ratio, and the AH Plus and EndoSeal MTA + PUI groups had similar remaining ratios. Calcium silicate-based sealers are advantageous for use canals subject to wet conditions due to an open apex or root canal perforation, as well as those with severe curvature and canals that are difficult to fill using pressure compaction. The clinical favorability and mechanical advantageousness of these properties have been demonstrated in earlier research [31]. However, the retrievability-related properties still require improvement prior to widespread clinical applications [9].

The limitations of this study include a relatively small sample size and incomplete standardization. Nonetheless, it makes a meaningful contribution by evaluating the efficiency of additional PUI use in the removal of EndoSeal MTA sealer.

## 5. Conclusion

EndoSeal MTA showed the largest amount of residual filling material after retreatment and may be more difficult to remove from the apical canal during canal root retreatment. The additional use of PUI can improve the efficiency of removal, especially in the middle and coronal thirds of the root canal.

## Figures and Tables

**Table 1:** Remaining ratio (%) in canals with an isthmus according to the root position in the AH Plus, EndoSeal MTA, and EndoSeal+PUI groups.

Location	AH Plus	EndoSeal MTA	EndoSeal MTA + PUI
Apical	7.46 (1.37-31.38)	45.14 (35.14-62.81)	30.13 (5.81-47.06)
Middle	8.41 (2.7-25.93)	22.94 (11.49-31.46)	3.19 (0.29-10.73)
Coronal	2.29 (0.79-8.32)	23.37 (15.08-32.06)	0.79 (0.14-5.86)
*p* value	0.149	< 0.001^∗∗^	0.002^∗∗^
*p* value [1]	1.000	0.003^∗∗^	0.057
*p* value [2]	0.270	0.003^∗∗^	0.002^∗∗^
*p* value [3]	0.278	1.000	0.772

The normality of the data distribution was tested using the Lilliefors test and the Shapiro-Wilk test. Variables are presented as median (interquartile range), and *p* values were computed using the Kruskal-Wallis *H* test due to the nonnormality of the variables. Post hoc analysis was performed using the Bonferroni correction, and the adjusted *p* values are presented as below: *p* value [1] computed between apical and middle. *p* value [2] computed between apical and coronal. *p* value [3] computed between middle and coronal. ^∗^*p* < 0.05, ^∗∗^*p* < 0.01, ^∗∗∗^*p* < 0.001.

**Table 2:** Remaining ratio (%) in canals without an isthmus according to the root position in the AH Plus, EndoSeal, and EndoSeal+PUI groups.

Location	AH Plus	EndoSeal MTA	EndoSeal MTA + PUI
Apical	11.57 (3.96-37.56)	18.97 (6.25-44.5)	1.26 (0.08-10.53)
Middle	1.93 (1.26-15.49)	3.43 (1.15-14.81)	0.04 (0-0.25)
Coronal	0.55 (0.18-1.21)	0.58 (0.03-5.09)	0.02 (0-0.4)
*p* value	< 0.001^∗∗^	0.001^∗∗^	0.026^∗^
*p* value [1]	0.065	0.059	0.054
*p* value [2]	< 0.001^∗∗^	< 0.001^∗∗^	0.063
*p* value [3]	0.090	0.588	1.000

The normality of the data distribution was tested using the Lilliefors test and the Shapiro-Wilk test. Variables are presented as median (interquartile range), and *p* values were computed using the Kruskal-Wallis *H* test due to the nonnormality of the variables. Post hoc analysis was performed using the Bonferroni correction, and the adjusted *p* values are presented as below: *p* value [1] computed between apical and middle. *p* value [2] computed between apical and coronal. *p* value [3] computed between middle and coronal. ^∗^*p* < 0.05, ^∗∗^*p* < 0.01, ^∗∗∗^*p* < 0.001.

**Table 3:** Comparisons of the remaining ratio between canals with and without an isthmus in the three groups.

Subgroup	Canal with an isthmus	Canal without an isthmus	*p* value
AH Plus	5.12 (2.51-14.2)	2.86 (1.49-7.85)	0.267
EndoSeal MTA	28.47 (21.25-32.29)	4.96 (2.55-10.75)	< 0.001^∗∗^
EndoSeal MTA + PUI	6.06 (2.16-14.89)	0.32 (0.07-1.44)	0.001^∗∗^
Subgroup	Coronal	Coronal	*p* value
AH Plus	2.29 (0.79-8.32)	0.55 (0.18-1.21)	0.015^∗^
EndoSeal MTA	23.37 (15.08-32.06)	0.58 (0.03-5.09)	< 0.001^∗∗^
EndoSeal MTA + PUI	0.79 (0.14-5.86)	0.02 (0-0.4)	0.015^∗^
Subgroup	Middle	Middle	*p* value
AH Plus	8.41 (2.7-25.93)	1.93 (1.26-15.49)	0.116
EndoSeal MTA	22.94 (11.49-31.46)	3.43 (1.15-14.81)	0.003^∗∗^
EndoSeal MTA + PUI	3.19 (0.29-10.73)	0.04 (0-0.25)	< 0.001^∗∗^
Subgroup	Apex	Apex	*p* value
AH Plus	7.46 (1.37-31.38)	11.57 (3.96-37.56)	0.325
EndoSeal MTA	45.14 (35.14-62.81)	18.97 (6.25-44.5)	0.019^∗^
EndoSeal MTA + PUI	30.13 (5.81-47.06)	1.26 (0.08-10.53)	0.006^∗∗^

The normality of the data distribution was tested using the Lilliefors test and the Shapiro-Wilk test. Variables are presented as median (interquartile range), and *p* values were computed using the Mann–Whitney *U* test due to the nonnormality of the variables. ^∗^*p* < 0.05, ^∗∗^*p* < 0.01, ^∗∗∗^*p* < 0.001.

## Data Availability

The data used to support the findings of this study are included within the article.
